# Heightened Virulence of *Yersinia* Is Associated with Decreased Function of the YopJ Protein

**DOI:** 10.1128/IAI.00430-21

**Published:** 2021-11-16

**Authors:** Chris A. Mares, Fernando P. Lugo, Mohammad Albataineh, Beth A. Goins, Irene G. Newton, Ralph R. Isberg, Molly A. Bergman

**Affiliations:** a Department of Microbiology, Immunology, and Molecular Genetics, The University of Texas Health Science Center at San Antonio, San Antonio, Texas, USA; b Department of Radiology, The University of Texas Health Science Center at San Antonio, San Antonio, Texas, USA; c Department of Biology, Indiana University, Bloomington, Indiana, USA; d Department of Molecular Biology & Microbiology, Tufts University School of Medicine, Boston, Massachusetts, USA; e Howard Hughes Medical Institute, Chevy Chase, Maryland, USA; University of Pennsylvania

**Keywords:** host-pathogen interactions, pathogenesis

## Abstract

Despite the maintenance of YopP/J alleles throughout the human-pathogenic *Yersinia* lineage, the benefit of YopP/J-induced phagocyte death for *Yersinia* pathogenesis in animals is not obvious. To determine how the sequence divergence of YopP/J has impacted *Yersinia* virulence, we examined protein polymorphisms in this type III secreted effector protein across 17 *Yersinia* species and tested the consequences of polymorphism in a murine model of subacute systemic yersiniosis. Our evolutionary analysis revealed that codon 177 has been subjected to positive selection; the Yersinia enterocolitica residue had been altered from a leucine to a phenylalanine in nearly all Yersinia pseudotuberculosis and Yersinia pestis strains examined. Despite this change being minor, as both leucine and phenylalanine have hydrophobic side chains, reversion of YopJ^F177^ to the ancestral YopJ^L177^ variant yielded a Y. pseudotuberculosis strain with enhanced cytotoxicity toward macrophages, consistent with previous findings. Surprisingly, expression of YopJ^F177L^ in the mildly attenuated *ksgA*^−^ background rendered the strain completely avirulent in mice. Consistent with this hypothesis that YopJ activity relates indirectly to *Yersinia* pathogenesis *in vivo*, *ksgA*^−^ strains lacking functional YopJ failed to kill macrophages but actually regained virulence in animals. Also, treatment with the antiapoptosis drug suramin prevented YopJ-mediated macrophage cytotoxicity and enhanced Y. pseudotuberculosis virulence *in vivo*. Our results demonstrate that *Yersinia*-induced cell death is detrimental for bacterial pathogenesis in this animal model of illness and indicate that positive selection has driven YopJ/P and *Yersinia* evolution toward diminished cytotoxicity and increased virulence, respectively.

## INTRODUCTION

Bacterial pathogens induce host cell death by various mechanisms and with downstream consequences that seem counterintuitive (1). At face value, the death of host cells, particularly cells of the innate immune system, should cripple the host immune response and allow pathogens to escape clearance. However, the opposite often occurs—the induction of host cell death can induce a host response that leads to pathogen removal (1). Mechanisms underlying this outcome are incompletely understood but likely include removing niches for intracellular replication or enhancing recruitment of highly activated phagocytes that then ingest and degrade the offending bacteria. Both host and bacterial factors are necessary for pathogen-induced host cell death, with caspase-1 being a notable player during macrophage pyroptosis (2) and required for host protection against pyroptosis-inducing pathogens (3, 4). Thus, pathogen-triggered cytotoxicity is a host defense strategy. But why do pathogens maintain the genes encoding the procytotoxicity factors in their genomes? The existence of such genes throughout the bacterial kingdom suggests that host cell death plays an important role in the persistence of individual species.

Three species of mammalian-pathogenic *Yersinia*, the gastroenteritis-causing Yersinia enterocolitica and Yersinia pseudotuberculosis and the plague-causing Yersinia pestis, have maintained such a procytotoxicity factor throughout their evolutionary history, in spite of significant genomic decay (5, 6). YopJ (so-called in Y. pestis and Y. pseudotuberculosis; called YopP in Y. enterocolitica) is one of the 6 to 8 type 3 secretion system (T3SS) effector proteins (*Yersinia* outer proteins [Yops]) injected by the bacteria into host cells (7–9). YopJ has been described as having ubiquitin-like protein protease (10), deubiquitinase (11, 12), and acyl transferase activity (13, 14), with acyl transferase activity requiring the host cell cofactor inositol hexaskisphosphate (15). In macrophages, YopJ activity inhibits mitogen-activated protein kinase (MAPK) and NF-κB signaling pathways, which in conjunction with TLR4 signaling results in host cell death (reviewed in reference 16), and recent evidence demonstrates that YopJ-induced phagocyte death occurs by a mechanism distinct from both apoptosis and pyroptosis but linked to necrosis (17). Regardless of the mechanism, the outcome is clear—YopJ has profoundly negative effects on macrophage viability.

However, exactly what YopJ does for *Yersinia* sp. *in vivo* is unclear. YopJ seems minimally important for virulence, at least compared with most of the other Yops, as YopJ^−^ Y. pestis, YopJ^−^ Y. pseudotuberculosis, and YopP^−^ Y. enterocolitica all display near-wild-type levels of virulence in various mouse models of acute illness (18–23). In contrast, there is evidence indicating that YopJ activity may actually diminish *Yersinia* virulence, as different isoforms of YopP/J can alter the degree of macrophage death and mouse illness following exposure to *Yersinia* sp. Y. pseudotuberculosis or Y. pestis expressing a hypersecreted variant of YopP showed enhanced cytotoxicity toward cultured macrophages but diminished virulence in mice (24, 25). Moreover, Y. pestis strain KIM also carries a hypercytotoxic YopJ variant, which when expressed in Y. pseudotuberculosis induced more macrophage cell death due to enhanced IκB kinase β (IKKβ) binding and thus reduced NF-κB signaling; the impact of this variant upon Y. pseudotuberculosis*-*caused illness in mice was not reported (26).

It is unknown if changes in YopP/J sequences occurred as a consequence of Darwinian evolution, but given that host-pathogen interactions can exert strong natural selection pressure upon both organisms (27), it seems likely that the YopJ-target protein interactions have been guided by evolutionary pressure. Evidence of selection at the codon level can be detected via phylogenetic analyses across multiple lineages to detect patterns of protein polymorphisms relative to divergence (28, 29). The advantage of this approach is that it can reveal loci affected by positive selection, even if the selection pressure is obscure. Such analyses of bacterial pathogen effector proteins have revealed signatures of molecular evolution, with individual codons showing evidence of directional selection (30, 31).

To address the hypothesis that YopP/J has been subjected to directional selection, we analyzed 17 sequences from multiple strains of each human-pathogenic *Yersinia* species for evidence of microevolution and evaluated how the different isoforms impacted the outcome of systemic subacute yersiniosis in a small animal model. Our studies reveal that some YopJ residues differing between the species have been subjected to evolutionary pressure. Reversion of a positively selected residue to the ancestral one yielded a YopJ isoform with enhanced cytotoxicity, but one that markedly attenuated Y. pseudotuberculosis virulence in animals. Conversely, the loss of YopJ due to catalytic inactivation or deletion rendered Y. pseudotuberculosis strains noncytotoxic but hypervirulent. Chemical inhibition of macrophage death during *in vivo* infection also enhanced Y. pseudotuberculosis virulence. Our results indicate that YopJ has evolved toward a diminished ability to induce macrophage cell death and that YopJ-induced death is detrimental for Y. pseudotuberculosis infection. This result may also suggest that some level of YopP/J-induced death is beneficial to the genus, perhaps by allowing persistence in an unknown reservoir.

## RESULTS

### YopJ has been positively selected in Y. pestis and Y. pseudotuberculosis for reduced cytotoxicity and enhanced virulence.

**FIG 1:**
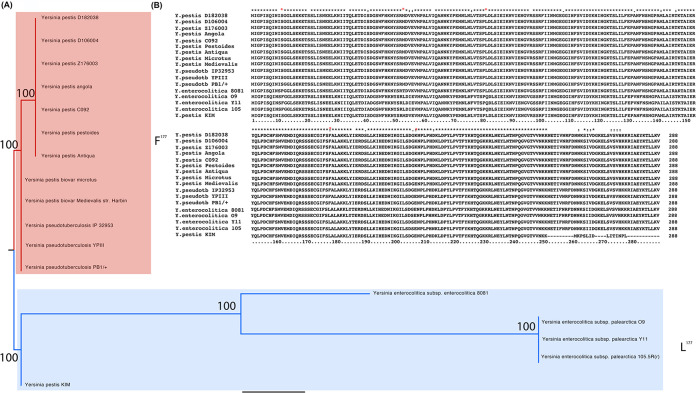
YopP/J is divergent between *Yersinia* species and shows evidence of positive selection. (A) The phylogeny of three *Yersinia* species (Y. enterocolitica, 3 strains; Y. pseudotuberculosis, 2 strains; and Y. pestis, 9 strains) generated using the YopJ primary sequences. Bootstrap support based on 1,000 replicates. Codon 177 was identified as under positive selection in a naive empirical Bayes (NEB) analysis, and amino acid polymorphism at that site is mapped onto the phylogeny of these *Yersinia* strains (“L” in blue and “F” in red). (B) Alignment of the relevant region of YopP/J homologs from these 14 *Yersinia* strains from the 3 human-pathogenic species. YopP/J was identified as having experienced positive selection at residue 177.

The *yopP/J* gene has been maintained in the genomes of all strains of human-pathogenic *Yersinia* species sequenced to date (5, 32, 33). This conservation is suggestive of an important role for the YopJ protein in natural settings. Although YopJ homologs are found in the genomes of all 3 human-pathogenic *Yersinia* species, sequence conservation differs across the length of the molecule. Some of the sequence polymorphisms group Y. pestis and Y. pseudotuberculosis to the exclusion of Y. enterocolitica (the expectation), while some other polymorphisms do not cleanly follow the predicted phylogeny of the organisms (Fig. 1A). We investigated whether or not these divergent residues show evidence of positive selection using a likelihood ratio test comparing support in the data for various models of evolution (either allowing for selection or imposing a nearly neutral model of evolution) (34, 35). This analysis identified sites under positive selection (that is, ancestral sequence reconstruction and the number and kind of differences at each residue suggested that the ratio of nonsynonymous to synonymous evolutionary changes [dN/dS] is >1) (see Table S1 and S2 in the supplemental material) and included residue 177, which is a leucine in Y. enterocolitica and Y. pestis KIM but a phenylalanine in the other Y. pestis homologs examined and all the Y. pseudotuberculosis YopJ homologs (Fig. 1B). Interestingly, the YopJ^F177L^ isoform was described recently to have enhanced activity relative to the YopJ^F177^ protein, promoting phosphorylation of IκB-α, increasing binding to IKKβ, and causing enhanced macrophage cytotoxicity (26).

**FIG 2:**
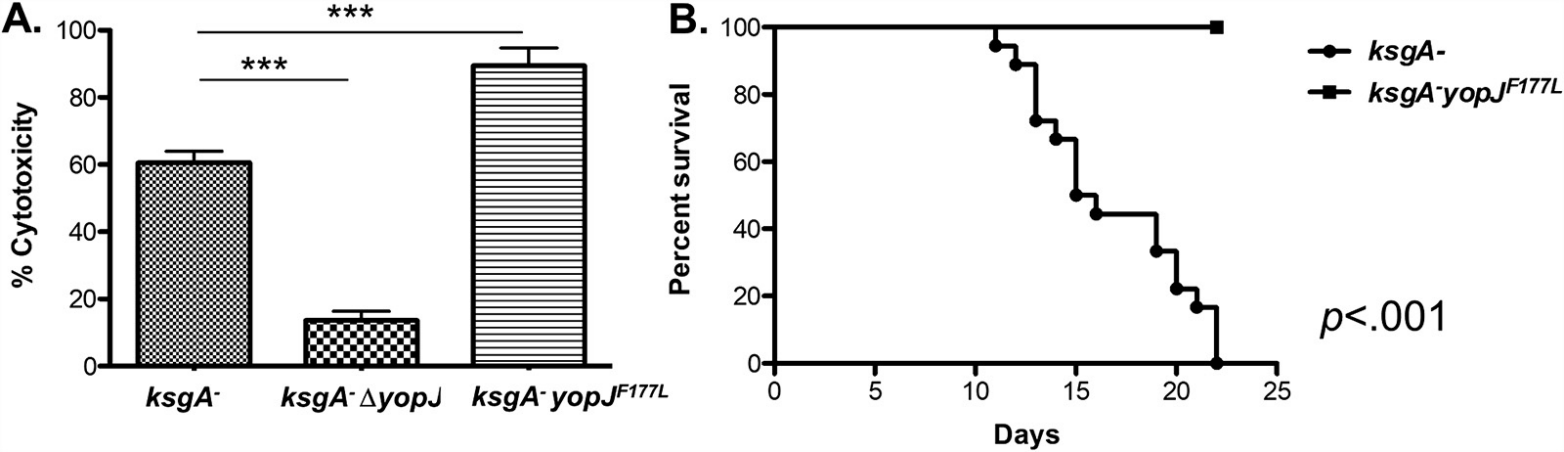
Ancestral version of YopP/J confers enhanced macrophage death and attenuated virulence. (A) Bone marrow-derived macrophages were exposed to *ksgA*^−^, *ksgA*^−^ *yopJ*, or *ksgA*^−^ *yopJ^F177L^* Y. pseudotuberculosis at an MOI of 100:1. LDH release was measured to determine the cytotoxicity of these strains in macrophages. (B) C57BL/6 mice were challenged intravenously with 1 × 10^3^ CFU of either *ksgA*^−^ (*n *= 12) or *ksgA*^−^ *yopJ^F177L^* (*n *= 11) Y. pseudotuberculosis. Morbidity and mortality were followed after challenge, and data shown represent the percent survival of each group. *In vivo* data are compiled from two independent experiments. Student’s *t* test was used to analyze BMDM infections. The Kaplan-Meier method was used to generate survival curves, and the log-rank test was used to calculate the significance (**, *P < *0.01; ***, *P < *0.005).

The existence of a hypercytotoxic YopJ variant allowed us to query if enhanced YopJ activity altered Y. pseudotuberculosis virulence in a small animal model of systemic subacute illness. Naive mice succumb rapidly to infection with virulent Y. pseudotuberculosis. Therefore, in order to better understand the phenotypes in this study, we utilized a strain deficient in KsgA that had been shown previously to have a slower replication rate than wild-type Y. pseudotuberculosis due to a loss of demethylation of 16S rRNA (36). We, and others, have also documented that this strain is attenuated *in vivo* (36, 37). Furthermore, we utilized the intravenous route of infection in order to investigate the role of YopJ after Y. pseudotuberculosis has disseminated to distal target tissues. As reported previously, YopJ^F177L^ shows increased cytotoxicity for macrophages relative to the YopJ^F177^ isoform (Fig. 2A). Using the attenuated strain as our baseline background strain (*ksgA^−^*), we observed that *ksgA^−^* bacteria expressing YopJ^F177L^ were more attenuated for virulence than the parental *ksgA^−^* strain, as demonstrated by the 100% survival rate of mice exposed to the *ksgA*- *yopJ^F177L^* strain (Fig. 2B). Increasing the inoculum dose did not reverse the complete attenuation of the strain (data not shown). These results indicate that the YopJ^F177L^-containing isoform attenuates virulence in the derived Y. pseudotuberculosis strains, correlating with the enhanced cytotoxicity conferred by this variant.

### Y. pseudotuberculosis strains lacking functional YopJ display increased virulence *in vivo*.

**FIG 3:**
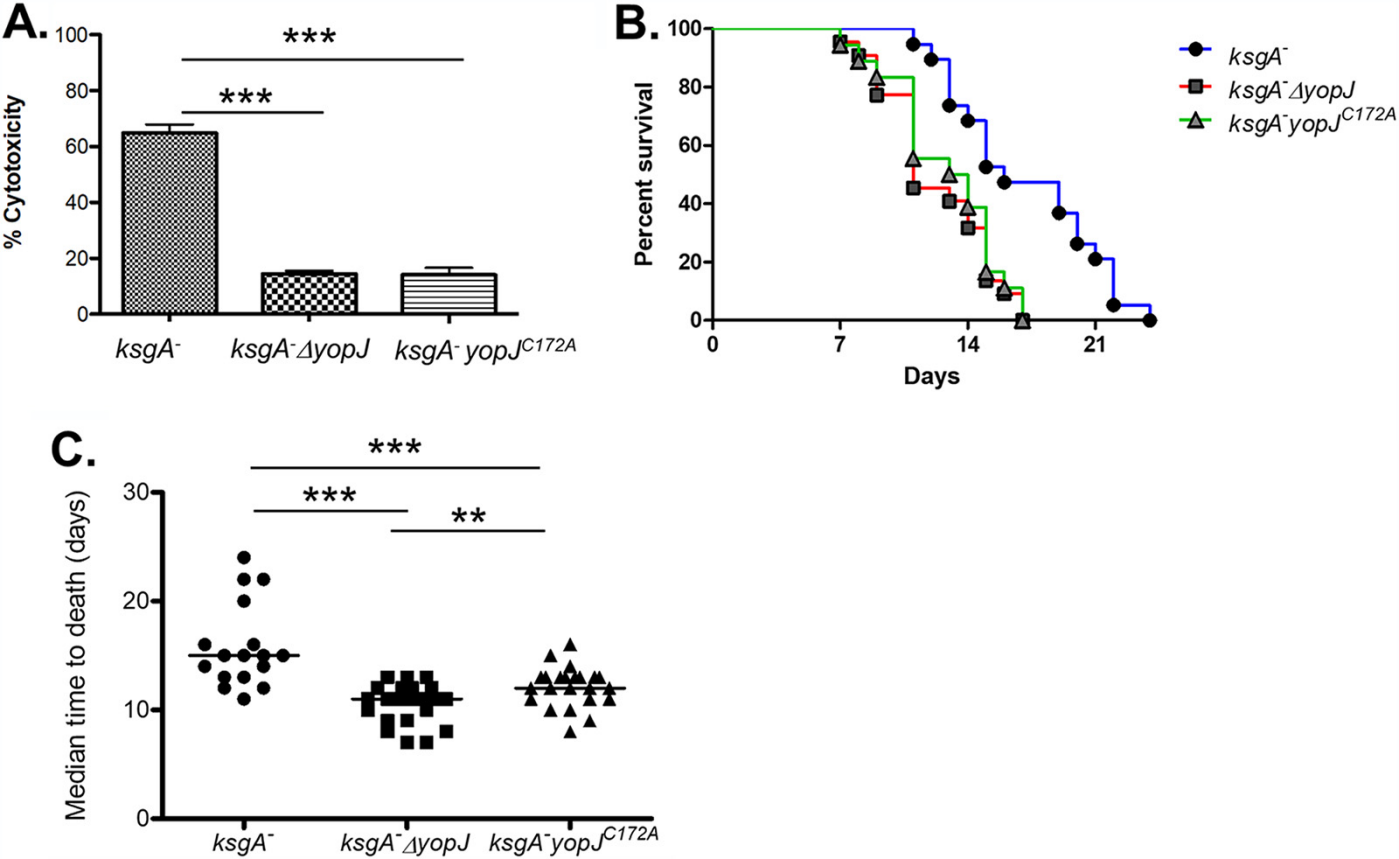
Loss of YopJ reverses the attenuation phenotype of *ksgA*^−^ Y. pseudotuberculosis. (A) Bone marrow-derived macrophages were infected with *ksgA*^−^, *ksgA*^−^ *yopJ*, or *ksgA*^−^ *yopJ^C172A^* Y. pseudotuberculosis at an MOI of 100:1 as described in the Materials and Methods section. One of four independent experiments is shown. LDH release was measured to determine the cytotoxicity of these strains in macrophages. (B) C57BL/6 mice were challenged intravenously with 1 × 10^3^ CFU of *ksgA*^−^ (*n *= 19), *ksgA*^−^ *yopJ* (*n *= 22), or *ksgA*^−^ *yopJ^C172A^* (*n *= 18) Y. pseudotuberculosis and monitored for survival (*ksgA*^−^ versus *ksgA*^−^ *yopJ*, *P* < 0.001; *ksgA*^−^ versus *ksgA*^−^ *yopJ^C172A^*, *P* < 0.001; *ksgA*^−^ *yopJ* versus *ksgA*^−^ *yopJ^C172A^*, *P* value not significant). (C) The median time until death was also assessed for each experiment. *In vivo* data were compiled from 4 independent experiments. The Student’s *t* test was used to analyze BMDM infections. The Kaplan-Meier method was used to generate survival curves, and the log-rank test was used to calculate the significance. The Mann-Whitney U test was used to compare median time to death (MTD) data (**, *P < *0.01; ***, *P < *0.005).

Given that a hypercytotoxic YopJ isoform attenuated Y. pseudotuberculosis illness in mice, we predicted that the absence of YopJ would enhance the virulence of the organism. To test this hypothesis, we constructed an isogenic *yopJ* deletion strain in the *ksgA^−^* background and confirmed that *ksgA^−^* Δ*yopJ* bacteria were noncytotoxic for bone marrow-derived macrophages (BMDMs) (Fig. 3A). Consistent with the negative correlation between cytotoxicity and virulence, mice inoculated with the *ksgA^−^* Δ*yopJ* strain died significantly sooner than those challenged with the *ksgA^−^* strain (Fig. 3B), with kinetics similar to the parental *ksgA^+^* strain (38). Confirming that the enhanced virulence phenotype of the *ksg^−^* Δ*yopJ* strain was due to the *yopJ* deletion, a strain rescued for the genomic Δ*yopJ* lesion by allelic exchange (*yopJ^repaired^*) caused a mortality rate identical to the parental *ksgA^−^* strain in animals (see Fig. S1 in the supplemental material).

The enzymatic activity of Y. pseudotuberculosis YopJ has been mapped to a triad of residues—histidine 109, glutamate 128, and cysteine 172 (10). We asked if the virulence phenotype of a strain carrying the catalytic *yopJ^C172A^* mutation mimicked that observed with the isogenic ΔyopJ strain. Similar to previous reports, the *ksgA^−^ yopJ^C172A^* strain was unable to kill cultured macrophages (Fig. 3A) (39). Like *ksgA^−^* Δ*yopJ* bacteria, *ksgA^−^ yopJ^C172A^* Y. pseudotuberculosis displayed increased virulence in animals relative to the parental strain (median time to death, day 11 versus 12, respectively). Interestingly, *ksgA^−^ yopJ^C172A^* bacteria and *ksgA^−^* Δ*yopJ* bacteria consistently displayed similar virulence and median times to death in multiple mouse survival assays (Fig. 3B and C). Taken together, these results indicate that either the absence of YopJ or the presence of inactive YopJ renders an attenuated strain of Y. pseudotuberculosis more virulent *in vivo.*

### The YopJ^F177L^ isoform attenuates the bacterial burden and increases host cell death *in vivo*.

**FIG 4:**
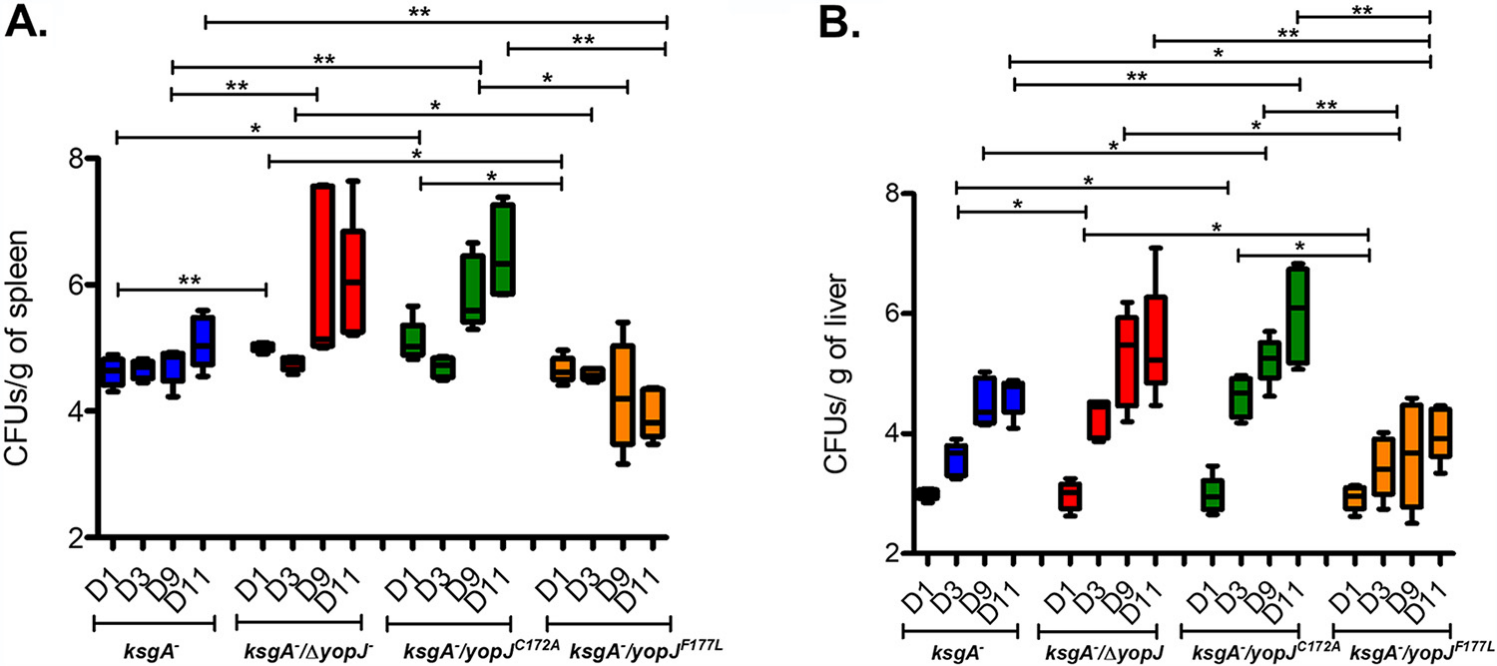
Y. pseudotuberculosis expressing YopJ^F177L^ show attenuated colonization *in vivo*. Liver (A) and spleen (B) were isolated at multiple time points from C57BL/6 mice after postchallenge (1.66 × 10^3^∼1.94 × 10^3^ CFU) with *ksgA*^−^, *ksgA*^−^ *yopJ*, *ksgA*^−^ *yopJ^C172A^*, or *ksgA*^−^ *yopJ^F177L^* Y. pseudotuberculosis. The tissues were homogenized, serially diluted, and plated onto LB plates. Burdens recovered in each tissue from each mouse were above the limit of detection. Data shown are from 5 mice/group and are representative of 2 independent experiments. The Mann-Whitney U test was used to compare CFU data (*, *P < *0.05; **, *P < *0.01).

To further understand the pathogenesis of the different YopJ-expressing strains *in vivo*, we studied the kinetics of bacterial colonization in the spleen and liver postchallenge, by examining burden at days 1, 3, 9, and 11 postchallenge. This analysis revealed two trends. First, at later time points, mice exposed to the *ksgA^−^ yopJ^F177L^* strains carried lower burdens of splenic and hepatic bacteria than mice exposed to the parental *ksgA^−^*, *ksgA^−^* Δ*yopJ*, and *ksgA^−^ yopJ^C172A^* strains (Fig. 4A and B). Second, the number of YopJ^F177^ and YopJ-deficient bacteria (both YopJ^−^ and YopJ^C172A^ mutants) generally increased over the 11-day time course in target organs (Fig. 4A, B), whereas the *ksgA^−^ yopJ^F177L^* bacteria were either slower to accumulate in tissues (liver, Fig. 4A) or diminished during this time frame (spleen, Fig. 4B). These results are consistent with YopJ triggering host immune responses that inhibit bacterial replication and/or remove bacteria from the tissue, while the absence of YopJ activity allows the bacteria to replicate unchecked in tissues.

In addition to causing macrophage cytotoxicity, YopJ can also inhibit macrophage production of cytokines (40–43), such that the increased mortality of mice exposed to *ksgA^−^* bacteria carrying either a null or inactive *yopJ* allele could result from a massive overproduction of cytokines, also known as a cytokine storm (44–46). However, we did not find any evidence indicating that the absence of functional YopJ triggers a mortality-inducing cytokine storm *in vivo* (see Fig. S2 in the supplemental material).

**FIG 5:**
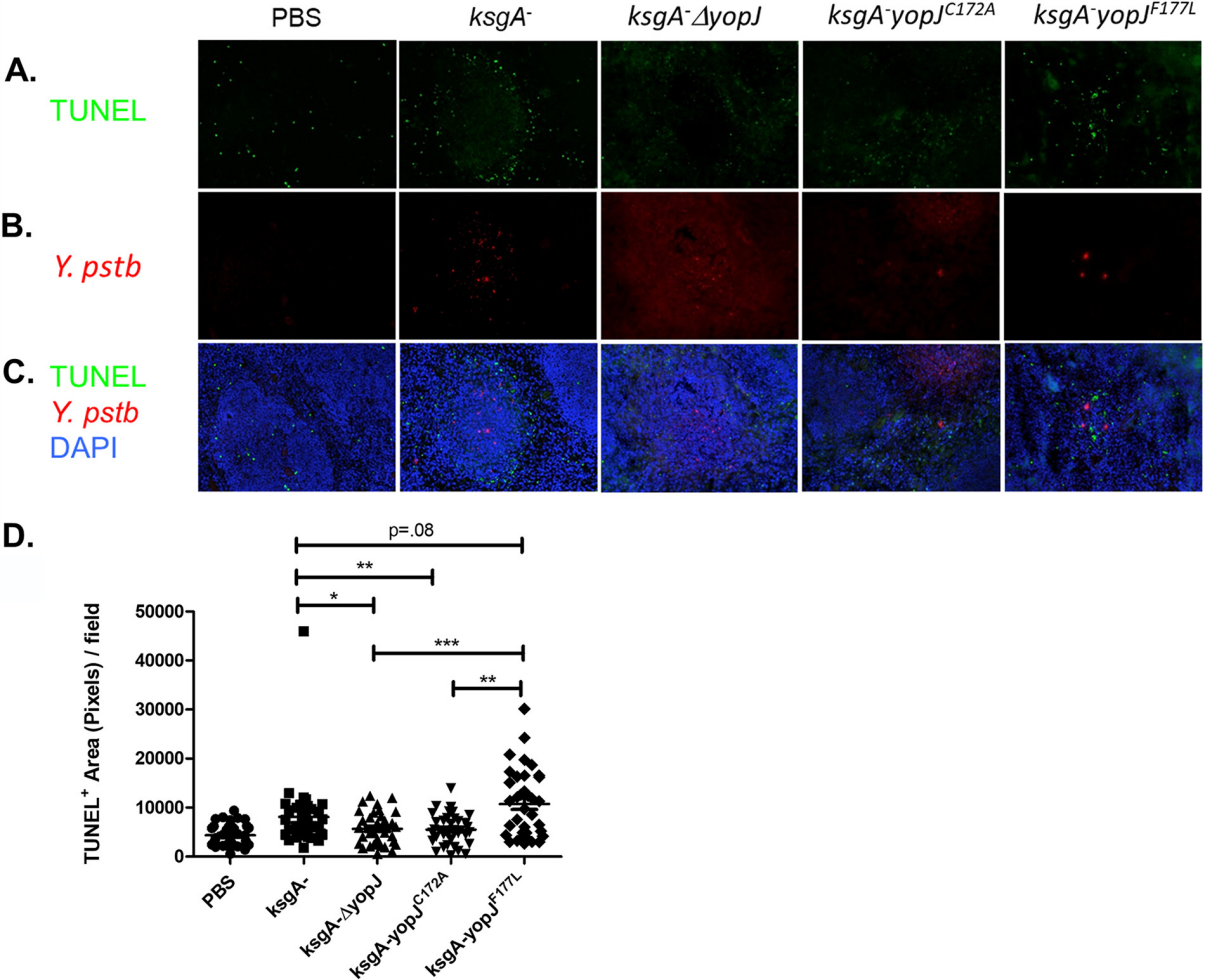
Ancestral YopP/J variant causes increased levels of cytotoxicity *in vivo*. Six- to 8-week-old female C57BL/6 mice were infected intravenously (1 × 10^3^∼1.26 × 10^3^ CFU) with strains of Y. pseudotuberculosis expressing *ksgA*^−^, *ksgA*^−^ Δ*yopJ*, *ksgA*^−^ *yopJ^C172A^*, or *ksgA*^−^ *yopJ^F177L^*. (A to C) Spleens were removed at day 3 postinfection and snap-frozen in OCT for TUNEL staining. Row A is showing TUNEL-only images at 200× and row B is showing staining of representative images for Y. pseudotuberculosis (rhodamine red X). Row C is depicting the merged images with DAPI (blue), TUNEL (fluorescein isothiocyanate [FITC]), and Y. pseudotuberculosis (rhodamine red X) at a total magnification of ×200. (D) Random images were captured (6 from each spleen, 3 mice per group per experiment), and TUNEL^+^ area (total pixels) per field was quantified using ImageJ software. D shows pooled results obtained from two independent experiments. The Mann-Whitney U test was used to compare TUNEL data (*, *P < *0.05; **, *P < *0.01; ***, *P < *0.005).

To determine if cell death levels *in vivo* recapitulated what we had observed with cultured macrophages exposed to the different Y. pseudotuberculosis strains, we used the terminal deoxynucleotidyltransferase-mediated dUTP-biotin nick end labeling (TUNEL) assay to detect dead mammalian cells in tissue sections from colonized mice. Mice were sacrificed at day 3 postinoculation for cell death evaluation to ensure equivalent burden levels between mice inoculated with the different strains (as observed in Fig. 4). This analysis revealed that at 3 days postinfection, spleens containing *ksgA^−^* (WT YopJF177) or *ksgA^−^ yopJ^F177L^* bacteria had increased levels of TUNEL^+^ cells compared with spleens containing the *ksgA^−^*ΔyopJ bacteria (Fig. 5 A to D), although the strain expressing catalytically dead YopJ did not perfectly phenocopy the *ksgA^−^* Δ*yopJ* strain (Fig. 5D). These results indicated that the increased survival of mice infected with *ksgA^−^ yopJ^F177L^* was significantly correlated with the amount of TUNEL staining present in their spleens at day 3 postinfection. Conversely, a decrease in the amount of TUNEL staining present in the spleens at this time point was associated with the early death of mice infected with either *ksgA^−^* Δ*yopJ* or with *ksgA^−^ yopJ^C172A^*.

### Suramin inhibits *Yersinia*-induced macrophage cytotoxicity and enhances virulence *in vivo*.

Our results suggest that YopJ-induced cytotoxicity is detrimental to Y. pseudotuberculosis pathogenesis, such that enhancing or inhibiting host cell death *in vivo* should impair or promote Y. pseudotuberculosis virulence, respectively. To test this hypothesis, we first considered if artificially increasing macrophage death in mice exposed to YopJ-null Y. pseudotuberculosis would rescue the mice from illness. Clodronate-containing liposomes are used commonly to deplete macrophages *in vivo*—depletion results because the clodronate induces global macrophage death (47); we confirmed that mice exposed to clodronate liposomes showed massive levels of apoptotic cells in tissues (data not shown). We inoculated mice with *ksgA*^−^ Δ*yopJ* Y. pseudotuberculosis and, 6 hours later, delivered a one-time injection of clodronate liposomones. The liposome-treated animals showed a slightly increased rate of mortality compared with mock-treated animals (see Fig. S3 in the supplemental materials), suggesting that the global depletion of macrophages masked any consequence of increased macrophage apoptosis.

**FIG 6:**
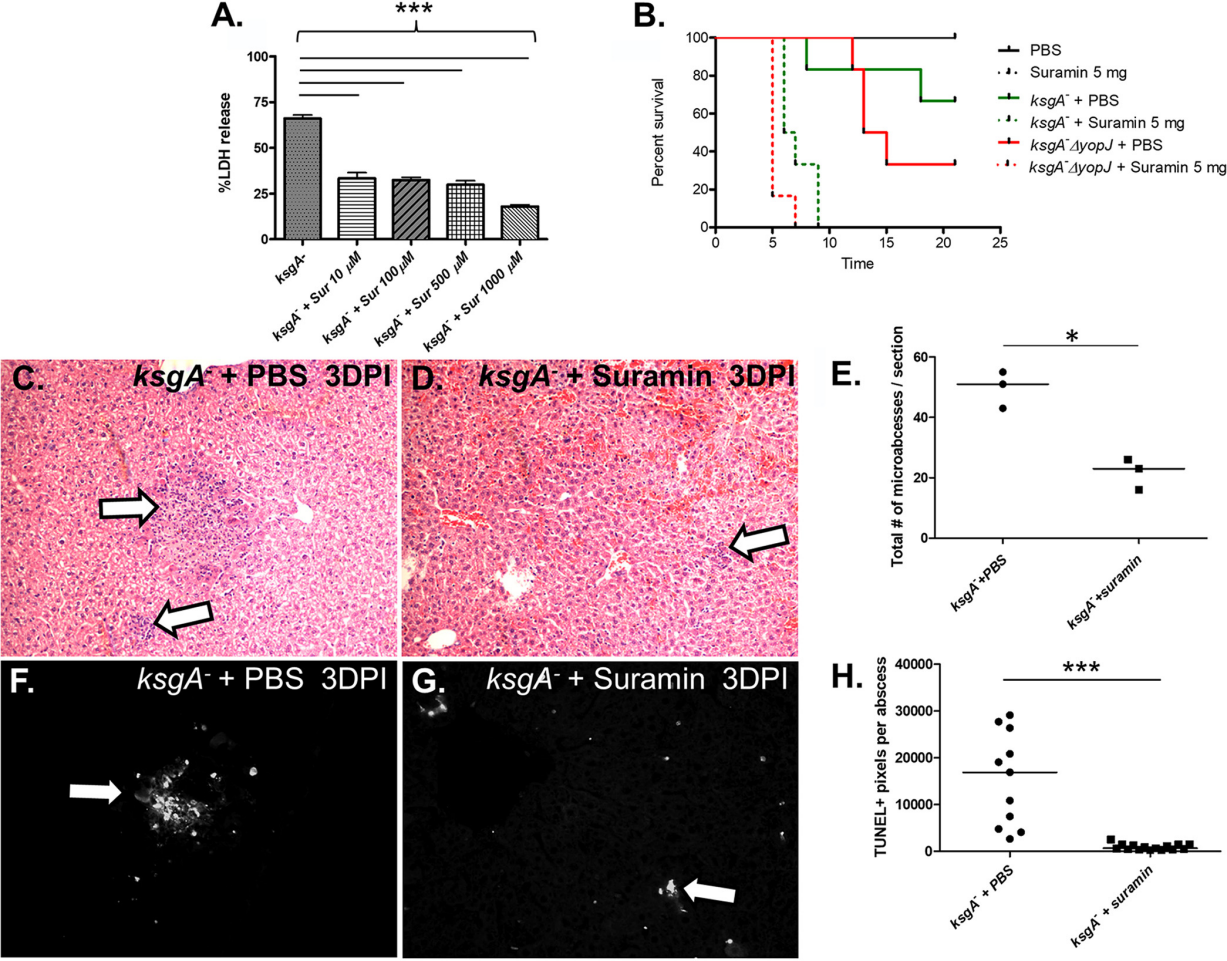
Suramin inhibits *Yersinia*-induced macrophage cytotoxicity and enhances virulence *in vivo*. (A) Bone marrow-derived macrophages from C57BL/6 mice were exposed to *ksgA*^−^ Y. pseudotuberculosis at an MOI of 100:1 in the presence or absence of suramin at different concentrations. LDH release was measured to determine macrophage cytotoxicity. (B) C57BL/6 mice (*n *= 3 to 6/group; repeated twice) were infected intravenously (1.08 × 10^3^∼1.38 × 10^3^ CFU) with various strains of Y. pseudotuberculosis in the presence or absence of suramin delivered intraperitoneally shortly after infection. Mice were weighed daily and monitored for signs of morbidity (*ksgA*^−^ + PBS versus *ksgA*^−^ + suramin, *P *= 0.0042; *ksgA*^−^ ΔyopJ + PBS versus *ksgA*^−^ ΔyopJ + suramin *P = *0.0005; *ksgA*^−^ + suramin versus *ksgA*^−^ ΔyopJ + suramin, *P *= 0.023). (C and D) C57BL/6 mice (*n *= 3 per group; repeated twice) were infected intravenously with Y. pseudotuberculosis *ksgA*^−^ (1 × 10^3^ CFU) and treated intraperitoneally with either 5 mg of suramin or with PBS. After 3 days, mice were sacrificed and livers were harvested and stained with H&E; large white arrows indicate representative lesions found in the sections. (E) Quantification of the number of microabscesses from the entirety of the H&E-stained liver sections (*n *= 3 per group). (F and G) TUNEL-stained liver sections are shown, with TUNEL^+^ lesions highlighted with white solid arrows. (H) Quantification of the degree of TUNEL positivity per microabscess (n = 4 to 6 microabscesses per liver section; *n *= 3 mice per group). The Kaplan-Meier method was used to generate survival curves, and the log-rank test was used to calculate the significance. The Student’s *t* test was used to analyze BMDM infections. The Mann-Whitney U test was used to compare TUNEL data (*, *P < *0.05; ***, *P < *0.005).

We next attempted to diminish host cell death levels *in vivo* to determine if it would enhance Y. pseudotuberculosis virulence. We turned to the apoptosis inhibitor suramin, which is a polysulfonated urea derivative capable of inhibiting death receptor-induced apoptosis and apoptosis-mediated liver damage (48, 49). Suramin blocked *Yersinia*-induced death of cultured macrophages in a dose-dependent manner (Fig. 6A), at the same levels shown previously to block hepatic cell cytotoxicity induced by death receptors CD95, TRAIL-R1, and TRAIL-R2 (48). To evaluate how suramin treatment affected Y. pseudotuberculosis virulence *in vivo*, mice were inoculated with bacteria and then injected with suramin 30 minutes to 1 hour postinoculation. Suramin had no effect on naive mice but dramatically increased mortality of mice exposed to *ksgA*^−^ bacteria (Fig. 6B). This effect was YopJ independent, however, as mice inoculated with *ksgA*^−^ Δ*yopJ* bacteria and injected subsequently with suramin showed a similar increase in time to death (Fig. 6B). Strikingly, gross pathological differences were apparent after examination of H&E-stained liver sections taken from *ksgA*-infected mice treated either with suramin or phosphate-buffered saline (PBS). The suramin-treated/infected mice had significantly fewer microabscesses visible in liver sections than PBS-treated/infected mice (Fig. 6C to E). Confirming that suramin successfully impaired host cell death, liver sections taken from suramin-treated mice at 3 days postbacterial inoculation showed significantly fewer TUNEL^+^ cells within abscesses than untreated mice (Fig. 6F to H). These results demonstrate that inhibition of host cell death dramatically worsens the survival outcome for mice experiencing Y. pseudotuberculosis-caused illness and strongly supports the premise that host cell death protects the host and checks the pathogen.

## DISCUSSION

Two surprising things emerged from our study. First, positive selection of YopP/J residues has occurred directionally with *Yersinia* evolution and has yielded an isoform with a diminished ability to induce macrophage cell death. Second, YopJ function is related inversely to bacterial pathogenesis—a hypercytotoxic YopJ variant attenuates *Yersinia* virulence in mammals, while nonfunctional YopP/J isoforms (or null alleles) confer hypervirulence in the context of subacute infection. The evolutionary forces that selected for bacteria with reduced YopJ activity or secretion are obscure, but this obscurity does not negate that positive selection of YopP/J codons has indeed occurred and these polymorphisms influence the degree of YopP/J-induced macrophage death and/or *Yersinia* virulence toward animals (Fig. 1) (see also references 24, 26, 50, 51). While we observed the YopJ phenotype in a model of subacute Y. pseudotuberculosis illness in inbred mice, we speculate that the selective niche was/is necessarily a warm-blooded wild animal. This speculation is based on the knowledge that YopJ and other Yops are not expressed at the ambient temperatures typical of fleas and soil (52, 53), which are the other niches known to harbor Y. pestis (54), such that YopJ likely evolved to target mammalian proteins.

**FIG 7:**
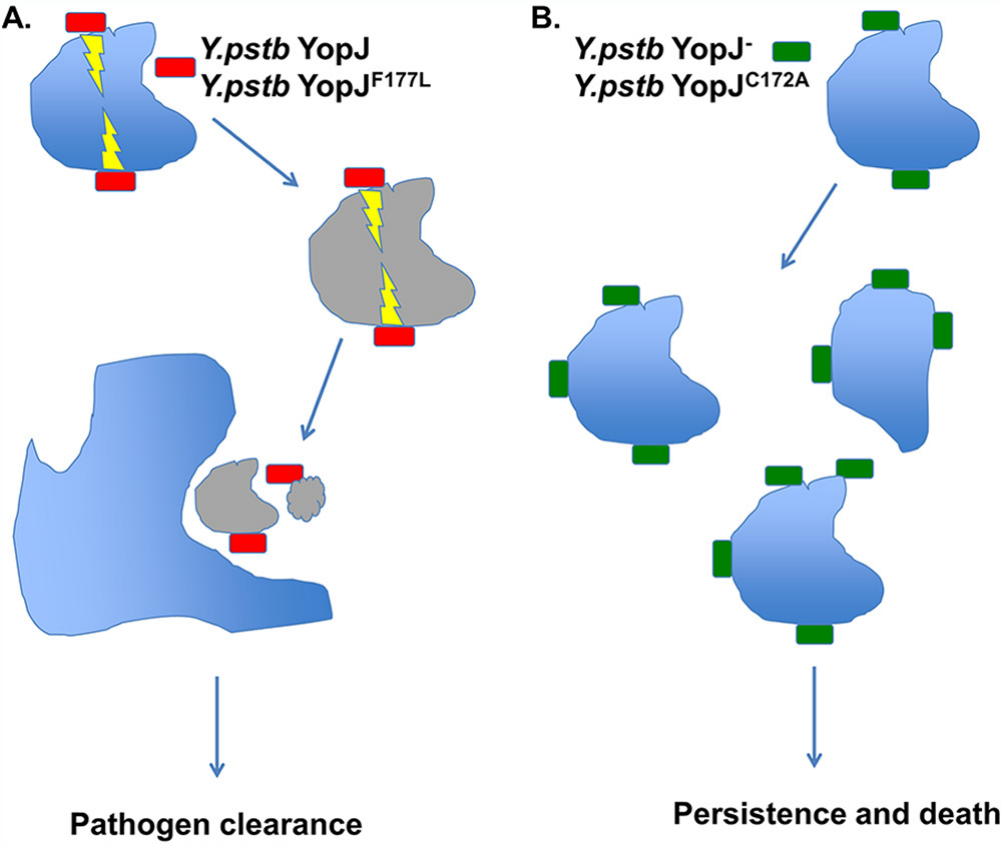
Proposed model to explain how different YopJ proteins lead to different *in vivo* outcomes following Y. pseudotuberculosis infection. (A) Y. pseudotuberculosis expressing functional YopJ (harboring either the wild type/positively selected F177 or the ancestral L177) attach to the host cell and inject YopJ, leading to host cell death. Efferocytosis of dead host cells by the neighboring activated phagocyte also removes those bacteria attached to dead host cells, leading to pathogen clearance. (B) Y. pseudotuberculosis expressing no or catalytically dead YopJ fail to induce host cell death, meaning the host cell would not be removed and bacteria could persist longer to cause illness. For either scenario, other factors, including host innate and adaptive immune responses (not shown), could also influence the balance between pathogen clearance and pathogen persistence.

How is YopJ activity diminishing Y. pseudotuberculosis virulence? Recent evidence has shown that YopJ can block the innate signaling interfering with transforming growth factor β (TGF-β)-activated kinase 1 (TAK1) in addition to interfering with NF-κB signaling and MAPK pathways (55–58). YopJ blockade of the aforementioned signaling events and in the presence of IR-domain-containing adapter-inducing interferon-β (TRIF) or tumor necrosis factor (TNF) signaling has been proposed to trigger receptor-interacting protein kinase 1 (RIPK1) and caspase-8 dependent apoptosis (57, 59, 60). Collectively, the above studies have shown that *Yersinia*-mediated RIPK1/caspase-8-mediated apoptosis can be protective for the host. Although the precise mechanism of *Yersinia*-induced cell death was not addressed in this study, our results similarly suggest that tailoring the ability of *Yersina* ability to induce host cell cytotoxicity may have an impact on the *in vivo* pathogenesis. We further speculate that the mechanism is similar to the one we observed previously in our studies of CD8^+^ T cell-mediated clearance of Y. pseudotuberculosis-associated target cells (37). In this model, bacteria expressing ancestral or hyperfunctional YopJ attach to the host cell and inject the YopJ protein, leading to host cell death. As dead host cells are efferocytosed rapidly by neighboring activated phagocytes, the attached bacteria are also removed in the process of efferocytosis, leading to pathogen clearance (Fig. 7A). In contrast, bacteria expressing no YopJ or catalytically dead YopJ would fail to induce host cell death, meaning the host cell would not be removed and bacteria could persist longer (Fig. 7B).

Given that the YopJ activity can be detrimental to *Yersinia* virulence, what then is the purpose of YopJ-induced macrophage death for *Yersinia*, when the absence of YopJ so clearly can increase *Yersinia* virulence during subacute illness? The continued maintenance of the *yopJ* gene in the Y. pseudotuberculosis and Y. pestis genomes, the latter of which has undergone marked decay (5, 6, 32), suggests the existence of a niche where YopJ function remains beneficial to Y. pestis. One possibility is that the beneficial or detrimental effects of YopJ on bacterial virulence depend on the host tissue environment. This speculation is supported by prior observations that YopJ-null Y. pseudotuberculosis bacteria have mild defects in virulence via oral inoculation but no defects via parental administration (18, 20, 24), suggesting that YopJ promotes extraintestinal dissemination of *Yersinia* sp. or is irrelevant in systemic tissues during acute infection, which is a hypothesis supported by recent observations that YopJ alters intestinal permeability and promotes *Yersinia* extraintestinal dissemination (24, 61). Other findings also point to a role for YopJ in the initial local infection site (25). Additionally, the role of YopJ in Y. pestis has been shown to be critical for dissemination and in triggering necroptosis rather than apoptosis. This study also showed that a ΔYopJ strain of Y. pestis led to host survival rather than death, as has been observed with ΔYopJ strains of Y. pseudotuberculosis (59, 62). Our findings with subacute systemic illness, however, demonstrate that YopJ actually hinders or impairs *Yersinia* virulence in systemic tissues. We speculate that YopJ activity serves as a self-regulating mechanism for *Yersinia* pathogenesis by allowing bacterial persistence in a resistant host or tolerant tissue, possibly in a relationship approaching commensalism or at least mild parasitism. In other words, YopJ may confer *Yersinia* an existence comparable to that observed with other chronic illness-causing pathogens—for example, Salmonella typhi, which persists in gallbladders and can be shed to disseminate to new environments and hosts. Another *Yersinia* Yop can also subvert host immune recognition to promote bacterial virulence; YopK inhibits inflammasome recognition of the *Yersinia* type III secretion system and thus prevents bacterial clearance (7). Although, it has been proposed that YopK acts by regulating T3SS translocation, rather than by direct activity toward target proteins within the host cytoplasm (63, 64). Additionally, there is one report demonstrating that the loss of YopT renders Y. enterocolitica hypervirulent (23). It may be that some Yops act to indirectly counter those Yops that promote virulence (i.e., YopE and YopH).

Our observations of mice exposed to noncytotoxic *ksgA*^−^ bacteria and of mice treated with the death-inhibitory chemical suramin during Y. pseudotuberculosis infection strongly suggest that host cell death is beneficial for host survival and detrimental to bacterial replication and/or existence. Our experiments show clearly that suramin-treated/*ksgA*-infected animals have a marked reduction in dead host cells (TUNEL^+^), suggesting that the diminished apoptosis plays at least a partial role in the increased susceptibility of suramin-treated animals to infection. Suramin has also been shown to inhibit cytotoxicity, decrease leukocyte infiltration, and dampen cytokine responses in other *in vivo* rodent models (65, 66). Therefore, it must be acknowledged that suramin has many potential uses and targets due to its complex pharmacology that may also be contributing to the results that we have observed with our *Yersinia* infection model (67). Our efforts to reduce host cell death levels with caspase inhibitors during *in vivo* infection were unsuccessful (data not shown), perhaps due to functional redundancy in the host cell death program components or the known consequence of increased necrosis/necroptosis following caspase inhibition (68).

YopP/J has homologues in other bacterial pathogens of animals (69), including Vibrio parahaemolyticus (VopP/A) (70, 71), Aeromonas salmonicida (AopP) (61), and Bartonella quintana strain Toulouse (YopP) (72). One interesting YopJ homologue is AvrA, which is found in numerous species, subspecies, and serovars of the genus Salmonella (33, 73). Like YopJ, AvrA is an deubiquitinase and acetyltransferase capable of modifying and inhibiting MAPKK and kinases in the Jun N-terminal protein kinase (JNK) and NF-κB signaling pathways (74, 75), leading to modulation of host cell inflammatory responses in the intestine (76). Interestingly, unlike YopJ, AvrA actually prevents apoptosis in host cells (75), suggesting that sequence differences between the two proteins may explain the divergence in function. Although AvrA does possess a leucine at the position that aligns with YopJ residue 177, suggesting that AvrA should be hyperfunctional relative to WT YopJ (YopJ^F177^), prior studies have shown that AvrA neither affects cytokine expression nor plays a role in macrophage killing when expressed by either Salmonella or *Yersinia* species (77). However, it should be noted that the *avrA* alleles used in these studies all possess a 3-nucleotide deletion that results in the absence of a leucine residue at position 139 (78, data not shown), which may explain why AvrA reportedly differs from YopJ in activity and function. However, regardless of the differences in sequence and tissue culture cell interactions, AvrA has two major features in common with YopJ—ability to attenuate virulence (75) and promote chronic infection (70) in mouse models of salmonellosis.

The ability of YopJ and AvrA to attenuate bacterial virulence is a feature typical of those YopJ homologues found in plant-associated bacteria. YopJ is a founding member of the YopJ/HopZ/AvrRxv superfamily, which is a family of T3SS-translocated proteins, expressed by both bacterial plant pathogens and symbionts, that are recognized by plant resistance (R) proteins during a plant defense response called the hypersensitive response (HR) (69). Induced host cell death is a critical feature of HR, and it serves to limit the spread of the bacterium beyond the initial infection site (79). YopJ homologues in plant-associated bacteria induce plant cell death in diverse hosts, including *Arabidopsis*, soybean, rice, tobacco, and pepper plants, among others, by targeting specific plant proteins for modification (69). The requirement of a common catalytic triad suggests the plant YopJ homologues have similar a enzymatic activity as YopJ. Supporting this suggestion, PopP2 from Ralstonia solanacearum has been shown to acetylate the *Arabidopsis* RRS1-R resistance protein (72), Xanthomonas campestris pv. vesicatoria AvrXv4 is a SUMO protease (80), and members of the HopZ family from Pseudomonas syringae have been shown to have protease or acetyltransferase activity (31, 81).

Another observation from our studies is reminiscent of plant R protein recognition of effectors. R proteins sense “altered self” in several ways, as follows: (i) direct modification of the R proteins by injected bacterial effector proteins, (ii) indirect recognition of effector protein-driven modifications of other host proteins, or (iii) indirect recognition of host cell molecules released in effector translocation or bacterial infection (82). We find it intriguing that bacteria expressing YopJ^C172A^ did not phenocopy the YopJ^−^ bacteria for mouse mortality and were attenuated slightly relative to the YopJ^−^ bacteria. This result has been seen before with YopO/YpkA, in a study of YopO/YpkA mutant strains in which Y. pseudotuberculosis-expressing nonfunctional YopO was attenuated, whereas a YopO-null strain was unaffected for virulence (83). We speculate that a nonfunctional YopJ protein may be perceived differently by the host than either a functional YopJ or the absence of YopJ, perhaps due to host recognition of the YopJ^C172A^ protein itself or recognition of YopJ^C172A^ binding to target host proteins. This recognition would then induce a cytosolic innate immune response to the foreign (albeit inactive) YopJ protein. This speculation remains to be addressed.

Phylogenetic and phylogenomic analyses are powerful approaches with which to identify signatures of molecular evolution in the genes and genomes of bacterial pathogens. A hallmark paper from Ma et al. determined that the C terminus of HopZ, the YopJ homologue found multiple P. syringae pathovars, has been under strong positive selection (31), providing further evidence that bacterial effectors that modulate host cell death are shaped by strong evolutionary forces. Interestingly, of the 14 pathovar HopZ sequences examined, all possessed a leucine at the position corresponding to YopJ 177 (31), suggesting that the expression of a highly functional HopZ is evolutionarily advantageous to multiple P. syringae pathovars. A recent phylogenomics study of 60 Chlamydia trachomatis strains detected signatures of positive selection in multiple T3SS effectors and also revealed that this selection is driving C. trachomatis evolution toward niche-specific adaptations (colonization of particular cell types or tissues) (30). It is almost certain that global or gene-specific analyses of molecular evolution in bacterial effector proteins will reveal evidence of positive selection, even if the selective forces and niches remain obscure.

Our work extends the findings of two recent *Yersinia* phylogenomic studies of note. Gu et al. examined the evolution and divergence of multigene families from 5 *Yersinia* strains (4 Y. pestis and 1 Y. pseudotuberculosis) and found evidence of functional diversities in genes associated with pathogenicity (84). While the results were suggestive of positive selection, the authors did not individually analyze Yops for microevolutionary signatures. In the second study, Cui et al. analyzed the genomes of 133 Y. pestis strains and identified 2,326 single-nucleotide polymorphisms (SNPs) that appear to have been fixed by neutral evolution rather than Darwinian selection (85). However, the authors analyzed only SNPs from the core genome of Y. pestis and not the accessory genome that includes the extrachromosomal plasmid carrying the *yop* genes, meaning that *yopJ* was not included in their analysis. Others have found at least 1 chromosomally encoded *Yersinia* protein that has undergone positive selection at the codon level, as an analysis of the OmpF porin sequence from 73 different *Yersinia* strains found positively selected residues in several surface-exposed OmpF domains (86). As such, our findings constitute the first description of diversifying selection in the accessory genome of any *Yersinia* species member. Although our analyses were performed on only a small subset of *Yersinia* YopP/J sequences, the site identified here, namely, codon 177, showed a significant level of positive selection, indicating the validity of our findings.

Our observations prompt the intriguing speculation that other Yops will also show evidence of molecular evolution. Given that the number of translocated *Yersinia* proteins is low (∼6 to 8 proteins), it seems likely that the importance of each individual effector is therefore increased, such that sequence divergence in response to any selective pressure would be greater. Microevolution within the sequences of other Yops, which generally function as provirulence factors, could result in Yops with increased functional abilities that confer increased virulence to individual strains or species. If true, it would present an interesting tug of war between provirulence Yops and antivirulence YopP/J, of which the outcome would likely depend on the niche.

Finally, the degree of positive selection in host proteins targeted by YopJ or other Yops is completely unknown. Recent observations of the molecular “arms race” between host and viral proteins demonstrated that the host antiviral protein protein kinase R (PKR) has been subjected to intense episodes of positive selection in primates in order to evade viral mimics of the PKR binding partner eIF2α (87). Regarding the latter, it is interesting to note that the activities of both YopJ and YpkA/YopO converge on a kinase of eIF2α (88). Further identification of YopP/J-specific target proteins and evaluation of sequence divergence through the primate lineage will reveal the degree of YopP/J-driven evolution of host immune response proteins.

## MATERIALS AND METHODS

### Bacterial strains and growth conditions.

Strains used in this study are indicated in Table S1, and primers used for strain construction are indicated in Table S2. Lysogeny broth (LB) and LB agar plates were made using the Lennox formulation (5 g/liter NaCl), and when necessary, the following selective antibiotics were included at the indicated concentrations: kanamycin, 30 µg/ml; ampicillin, 100 µg/ml; irgasan, 1 µg/ml; and chloramphenicol, 20 µg/ml. The Y. pseudotuberculosis strain YPIII pIB1 was the parent of all strains described here. The *ksgA*^−^ strain has been described and used by us previously (36, 89) and contains a kanamycin-resistance-encoding transposon inserted into the *ksgA* gene. To generate the *ksgA*^−^ Δ*yopJ* strain, we used a previously described suicide vector construct, pCVD442-Δ*yopJ* (kind gift of Joan Mecsas, Tufts University), in which the upstream and downstream DNA flanking *yopJ* open reading frame was fused together with only the first and last codon of *yopJ* remaining (90). Introduction of this unmarked *yopJ* deletion allele into the *ksgA*^−^ genome was accomplished via allelic exchange, using pCVD442-encoded ampicillin and sucrose resistance as the selectable and counterselectable markers, respectively. Briefly, the suicide vector was transferred from Escherichia coli strain SM10λpir to Y. pseudotuberculosis strain *ksgA*^−^ by conjugation, and ampicillin-resistant/irgasan-resistant integrants were selected. Integrants were grown overnight in the absence of plasmid selection, and then dilutions of the culture were placed on counterselection agar plates containing 10% sucrose and lacking salt. Sucrose-resistant colonies were confirmed to be ampicillin sensitive and the presence or absence of the Δ*yopJ* determined by PCR screening. To generate *yopJ* alleles carrying mutations in codons 172 and 177, a fragment of the *yopJ* gene was first cloned by PCR into pACYC184, and then site-directed mutagenesis was performed using long-range PCR with primers containing the mutant sequence and Dpn to remove the vector template. After the sequence change was confirmed, the mutated *yopJ* sequence was transferred to pCVD442, the allelic exchange was performed as described above, and the presence or absence of the mutant *yopJ* codons was determined by sequencing amplified genomic DNA. Bacteria were grown for cultured macrophage experiments as follows. After 2 days of growth on LB agar plates from glycerol stocks, single colonies from the indicated strains were inoculated into 2-ml LB broth and incubated at 26°C for 15 to 18 hours with rotation. Overnight cultures were back-diluted to an estimated optical density at 600 nm (OD_600_) of 0.2 into low-Ca^2+^ medium (2XYT broth containing 20 mM Ca^2+^ chelator sodium oxalate and 20 mM MgCl_2_) and then grown with constant rotation for 1.5 hours at 26°C and 1.5 hours at 37°C. Cultures were then adjusted to the desired concentration in macrophage media and used for macrophage challenges as described below. Bacteria were grown for mouse experiments as follows. After 2 days of growth on LB agar plates from glycerol stocks, single colonies were inoculated into 2-ml LB broth and incubated at 26°C for 15 to 18 hours with rotation. Overnight cultures were then adjusted to the desired concentration in PBS and used for mouse challenges as described below (after removing a sample for accurate determination of CFU/ml).

### Codon-level selection analysis of YopJ sequences across multiple *Yersinia* lineages.

To investigate the evolutionary history of YopJ, orthologs were found by using reciprocal blast searches across 15 sequenced genomes, including extrachromosomal elements and 2 individual *yopJ* loci (NC_003131, NC_003132, NC_003134, NC_003143, NC_004088, NC_004838, NC_005810, NC_005813, NC_005814, NC_005815, NC_005816, NC_006153, NC_006154, NC_006155, NC_008118, NC_008119, NC_008120, NC_008121, NC_008122, NC_008149, NC_008150, NC_008791, NC_008800, NC_009377, NC_009378, NC_009381, NC_009704, NC_009705, NC_009708, NC_010157, NC_010158, NC_010159, NC_010465, NC_010634, NC_010635, NC_014017, NC_014022, NC_014027, NC_014029, NC_015224, NC_015475, NC_017153, NC_017154, NC_017155, NC_017156, NC_017157, NC_017158, NC_017159, NC_017160, NC_017168, NC_017169, NC_017170, NC_017263, NC_017264, NC_017265, NC_017266, NC_017564, and NC_017565). Protein sequences were aligned using ClustalW, and nucleotide sequences for each YopJ ortholog were aligned based on their amino acid translation (using in-house scripts). These nucleotide alignments were used as input to RAxML (84), where maximum likelihood phylogenies were generated (GTRGAMMA model of substitution). A considerable amount of change in the YopJ homologs has occurred during the evolution of these pathogens; some of these changes alter protein encoding (dN, nonsynonymous nucleotide substitutions) while some do not (dS, synonymous substitutions). Evidence of adaptative selection was found across the aligned ortholog set by calculating the dN/dS in pairwise comparisons of orthologs. In order to identify those YopJ residues under selection and the direction and strength of selection at the codon level, the frequency of nonsynonymous and synonymous changes and their ratio (dN/dS or ω) were calculated using phylogenetic analysis by maximum likelihood (PAML v4.7) software using the Goldman and Yang amino acid substitution model (codeML) and these same nucleotide alignments (85). To increase the sensitivity of our analysis, we also calculated variable dN/dS (ω) over all sites (models M7 versus M8) and used the likelihood ratio test to identify statistically significant evidence for diversifying selection (χ^2^ = 14; df = 2; *P* < 0.01). In-house Perl scripts were used to parse the codeML output files to identify residues predicted to have undergone diversifying selection in YopJ (using maximum likelihood ratio tests before analyzing the results).

### Derivation of bone marrow macrophages.

Bone marrow-derived macrophages (BMDMs) were generated by culturing bone marrow cells harvested freshly from the femurs and tibias of C57BL/6 mice in supplemented Dulbecco’s modified Eagle’s medium (DMEM) as described previously (37). Macrophages were harvested on day 6 of the derivation protocol and seeded at a density of 5 × 10^4^ cells/100 µl/well of a 96-well plate in supplemented DMEM lacking antibiotics, and they were allowed to adhere to the plastic overnight. Occasionally, at day 6, macrophages were cryofrozen in 5% DMSO and 95% fetal bovine serum (FBS) at a concentration of 1 × 10^7^ cells/ml and subsequently thawed for seeding.

### Macrophage infections, treatments, and cytotoxicity assay.

Y. pseudotuberculosis, grown as described above, was added to adherent macrophages at a multiplicity of infection (MOI) of 100:1 in DMEM without phenol red + 1% FBS for 45 minutes to 1 hour. Gentamicin was added to a final concentration of 100 µg/ml, and the cells were allowed to incubate an additional 2 hours. For suramin experiments, BMDMs were first pretreated with various concentrations of suramin (Sigma, St. Louis, MO) for 1 hour and then exposed to bacteria at an MOI of 100:1 in the presence or absence of suramin at the indicated concentrations. Macrophage death was evaluated by detection of the cytoplasmic enzyme lactate dehydrogenase (LDH) in the culture supernatants similarly as described previously (37). Briefly, macrophages were spun down at 250 × *g* for 2 minutes, and 50 µl of the supernatant was carefully removed from each well, transferred to a new 96-well plate, and assayed immediately for the presence of LDH using the Cytotox 96 assay kit (Promega, Madison, WI) according to the manufacturer’s recommendations. Note that control cells were treated with 1% Triton X-100 to liberate cytoplasmic contents at approximately 15 minutes prior to supernatant harvest. A total of 50 µl of the reconstituted substrate solution [containing 2-(4-Iodophenyl)-3-(4-nitrophenyl)-5-phenyl-2H-tetrazolium chloride (INT)] was added to the harvested supernatants, the reaction was incubated at room temperature in the dark for 30 minutes, then the enzymatic conversion of INT into a red formazan product was terminated by addition of 50-µl stop solution (1 M acetic acid), and excess bubbles in each well were punctured. Absorbance was measured at 490 nm in a Synergy H4 plate reader (BioTek, Winooski, VT). Percent cytotoxicity was calculated as follows: 100× [(experimental release − effector T cell spontaneous release − target cell spontaneous release)/(target cell maximum release − target cell spontaneous release)].

### Mouse infections and treatments.

Six- to 8-week-old female C57BL/6 mice were purchased from the National Cancer Institute colony at Charles River Laboratories. Mice were allowed to rest for 7 days after arrival and then infected intravenously via tail vein injection with a 200-µl inoculum containing between 3.5 × 10^2^ and 2 × 10^3^ CFUs in PBS of the indicated Y. pseudotuberculosis strain. Mice were then inspected visually for signs of illness, including lethargy and piloerection, and weighed daily to monitor the disease progression. For suramin experiments, mice were infected as before and then injected intraperitoneally once with 5-mg suramin + 400 µl PBS or 400 µl PBS alone 30 minutes to 1 hour postinfection. For clodronate experiments, clodronate-containing liposomes were prepared as described below, and then 200 µl of liposomes were injected intravenously at 6 hours postinfection. All animal experiments were performed in accordance with the NIH Guide for the Care and Use of Laboratory Animals and were approved by the UTHSCSA Institutional Animal Care and Use Committee.

### Preparation and characterization of clodronate liposomes.

Liposomes containing dichloromethylene bisphosphonate (clodronate) or control empty liposomes containing phosphate-buffered saline (PBS) were prepared according to reported protocols with slight modifications (47, 91). In 2 500-ml round-bottom flasks, 96-mg cholesterol (Calbiochem, San Diego, CA) was added and dissolved with 10-ml chloroform (high-performance liquid chromatography [HPLC] grade; Fisher Scientific, Fair Lawn, NJ). Then, 4.14 ml of egg phosphatidylcholine in chloroform (25 mg/ml; Avanti Polar Lipids, Alabaster, AL) was added. Lipid films were formed following the removal of chloroform by rotary evaporation. The lipid films were placed under vacuum for 1 h to remove residual chloroform. The lipid films were rehydrated with 12 ml of 0.7 M clodronate (Sigma-Aldrich, St. Louis, MO) prepared in degassed sterile water for injection (pH adjusted to 7.0) (Hospira, Lake Forest, IL) or in degassed Dulbecco’s PBS (pH 7.4) (Invitrogen, Carlsbad, CA). The flasks were swirled until the lipids were in suspension. The lipid suspensions were kept at room temperature for 2 h with occasional swirling, sonicated in a water bath for 4 minutes (Branson, Danbury, CT), and then kept at room temperature for 2 h. The liposome samples were then extruded through polycarbonate filters (2 µ, 2 times; 1 µ, 4 times) using an extruder (Lipex, Northern Lipids, Vancouver, Canada) and collected in a new tube. After the liposome samples were flushed with argon, the samples were stored overnight at 4°C. The next day, the liposomes were transferred to microcentrifuge tubes and centrifuged in an Allegra 21R centrifuge (Beckman Coulter, Brea, CA) at 15,300 rpm for 30 minutes at 10°C. The supernatants were removed and the pellets resuspended with degassed PBS. The samples were washed 4 times. After the last wash, the liposome pellets were resuspended in degassed PBS to a final volume of 4.8 ml, flushed with argon, and stored at 4°C until needed. Liposomes were used for the study within 2 weeks of preparation. Liposome diameter was measured at 488 nm with a DynaPro dynamic light scattering system (Wyatt Technology, Santa Barbara, CA). The diameters for clodronate liposomes and control PBS liposomes were 968 ± 28 nm and 790 ± 41 nm, respectively. Phospholipid content was measured using a phosphatidylcholine colorimetric assay kit (Cayman Chemical, Ann Arbor, MI) and determined to be 14.5 mg/ml for the clodronate liposomes and 15.4 mg/ml for the PBS liposomes. The amount of liposome-encapsulated clodronate was determined to be 12.7 mg/ml (47). In pilot experiments, clodronate liposomes were confirmed to induce macrophage death by TUNEL staining in spleens of noninfected mice (data not shown).

### Organ bacterial burden assay.

At the noted times postinfection, mice were sacrificed by cervical dislocation; spleens and livers were removed aseptically, transferred to sterile PBS from mice at various time points after infection, and weighed. Tissues were disrupted mechanically using a tissue homogenizer (Omni, Marietta GA) and serially diluted in sterile PBS, and 100 µl of the dilutions was plated onto nutrient LB agar plates. After 48 hours at 26°C, bacterial colonies were enumerated and CFU normalized to the weight of tissue in grams. Note that as each animal may have had an organ weight different from the other animals, the CFU values at or below the limit of detection (10 CFU per ml of organ homogenate) were occasionally different between individual animals. The limit of detection in the spleen was 90 CFU/g/spleen or 1.95 log CFU and in the liver was 22 CFU/g/liver or 1.33 log CFU.

### Cytokine analysis.

The cytokine analysis was performed on serum and tissue homogenates from day 3 and day 9 postinoculation. Briefly, blood was collected via cardiac puncture and allowed to clot for 30 minutes. Serum was collected after centrifugation at 7,000 rpm for 7 minutes and stored at −80°C. Cytokines were assayed using the mouse cytokine 20-plex panel by adhering strictly to the manufacturer’s protocol (Life Technologies, Carlsbad CA). Cytokine levels were detected using a Bio-Plex 200 system and analyzed using Bio-Plex manager 6.0 software (Bio-Rad, Hercules CA).

### TUNEL assay.

Spleens were harvested at 3 days postinfection and were snap-frozen in the cyrostat sectioning medium Tissue-Tek O.C.T. (Sakura, Torrance CA). Livers were removed and placed in 10% buffered formalin for at least 3 days and then sent to the UTHSCSA Histopathology Laboratory to be embedded in paraffin and cut into 4-µm sections. Some slides with liver sections were stained with hematoxylin and eosin and others were processed for TUNEL staining with the ApopTag kit as outlined below. OCT-frozen spleen sections were cut with a cryostat (Thermo Scientific Shandon, Waltham MA), placed on slides, and allowed to air dry overnight. The slide-affixed tissue sections were then fixed in acetone for 20 seconds at room temperature, allowed to dry, then wrapped in foil, and stored at −80°C. Subsequently, sections were stained for nicked DNA to label TUNEL^+^ cells, using the ApopTag kit with included buffers (EMD Millipore, Billerica, MA). Briefly, slides were removed from −80°C and allowed to warm up to room temperature. Slides were then fixed in 1% paraformaldehyde for 10 minutes at room temperature and washed in PBS twice. Tissue sections were not allowed to dry after this point. Tissue sections were then circled with a hydrophobic PapPen (Kiyota International, Elk Grove Village, IL), and equilibration buffer was incubated on the section for at least 10 seconds. Terminal deoxynucleotidyl transferase (TdT) enzyme solution (10% enzyme + 20% PBS + 70% reaction buffer) was applied for 60 min in a humidity chamber at 37°C. Stop solution was then applied for 10 minutes at room temperature followed by three washes with PBS. A solution containing anti-digoxigenin fluorescein antibody (53% blocking solution + 47% anti-digoxigenin fluorescein conjugate) was applied and allowed to incubate for 30 minutes in a dark humidity chamber. Slides were then protected from light as much as possible from this point forward. Slides were then washed three times in PBS and mounted with ProLong Gold antifade reagent containing 4′,6-diamidino-2-phenylindole (DAPI) (Life Technologies, Carlsbad, CA). Sections from a given experiment were stained on the same day.

### Microscopy and image capture.

Stained tissue sections were visualized using either an EC Plan-Neofluar 10× or Plan-Apochromat 20×/0.8 objective on a Zeiss AxioImager Z1 epifluorescence microscope (Carl Zeiss, Thornwood, NY). Images were captured using a Zeiss AxioXam MRm Rev3 and/or MRc cameras and analyzed using Zeiss AxioVision release 4.5 software. Sections from a given experiment were imaged on the same day with the same exposure times for image acquisition to ensure consistency between samples. ImageJ (92) was used to measure the pixel intensity in each captured image.

### Data and statistical analysis.

Prism 5 (GraphPad Software, La Jolla CA) was used for graphing and statistical analysis. Survival curves were estimated using the Kaplan-Meier method and significance calculated using the log-rank test. The nonparametric Mann-Whitney U test and unpaired Student’s *t* test were used to determine statistical differences between groups of data from animal and tissue culture experiments, respectively. Image quantification data were evaluated for significance using the Mann-Whitney U test.
