# Supplementary material for: Heightened Virulence of Yersinia Is Associated with Decreased Function of the YopJ Protein
Source: Infect Immun. 2021 Nov 16;89(12):e00430-21. doi: 10.1128/IAI.00430-21 (PMC8594599; doi:10.1128/IAI.00430-21)

SUPPLEMENTAL FIGURE LEGENDS

S1. Repair of *yopJ in the ksgA*$^{-}$*ΔyopJ* strain restores the original phenotype. A. RAW macrophages were infected with either *ksgA*$^{-}$, *ksgA*$^{-}$*ΔyopJ*, or *ksgA*$^{-}$*yopJ*^{repaired} *Y. pseudotuberculosis* strains at an MOI of 100:1. LDH release was measured to determine the cytotoxicity of these strains in macrophages. (**B**. and **C.**) C57BL/6 mice were intravenously challenged with *ksgA*$^{-}$, *ksgA*$^{-}$*ΔyopJ* or *ksgA*$^{-}$*ΔyopJ*^{repaired} *Y. pseudotuberculosis*. Morbidity and mortality was followed after infection and data shown represents the percent survival and weight loss of each group (*n*=5 mice per group).

S2. A cytokine storm does not characterize illness caused by *ksgA-ΔyopJ* bacteria. C57BL/6 mice were inoculated intravenously (1~1.26x10^{3} CFU) with *ksgA*$^{-}$, *ksgA*$^{-}$*ΔyopJ*, or *ksgA*$^{-}$ *yopJ*^{C172A} *ksgA*$^{-}$ *yopJ*^{F177L} bacteria. Serum was collected from each mouse at day 9 post inoculation and was assayed for cytokines using a cytokine multiplex assay.

S3. Systemic clodronate liposome induced macrophage apoptosis in vivo does not protect mice from *Y. pseudotuberculosis* infection. 6-8 week old female C57BL/6 mice were infected intravenously (1x10^{3} CFU) with *ksgA*$^{-}$*ΔyopJ Y. pseudotuberculosis* and left untreated or treated with either PBS containing liposomes or clodronate containing liposomes at 6 hours post infection. Morbidity and mortality was followed after infection and data shown represents the percent survival of each group (*n*=6 mice per group). The Kaplein-Meier method was used to generate survival curves and the log-rank test was used to calculate the significance (*ksgA*$^{-}$*ΔyopJ* vs. *ksgA*$^{-}$*ΔyopJ* + PBS, n.s.;

ksgA$^-$ΔyopJ vs. *ksgA$^-$ΔyopJ* + clodronate, *p*=.064; *ksgA$^-$ΔyopJ* + PBS vs. *ksgA$^-$ΔyopJ* + clodronate, *p*=.065).

Table 1. ***Y. pseudotuberculosis*** **(YPIII pIB1) strains used in this study**

Strain	Strain name	Reference
ksgA⁻	500	[80]
ksgA⁻ΔyopJ	MB153	This study
ksgA⁻yopJ^{C172A}	MB219	This study
ksgA⁻yopJ^{F177L}	MB254	This study

Supplementary Table 1. Bayes Empirical Bayes results for positively selected sites in YopJ (*: P>95%; **: P>99%)

position	Residue	Prob (ω > 1)	Mean ω	Standard deviation
10	I	0.544	5.537	4.754
11	S	0.998**	9.843	1.001
20	S	0.938	9.291	2.394
33	T	0.790	7.904	3.852
40	S	0.581	5.891	4.715
52	M	0.999**	9.851	0.965
54	V	0.791	7.920	3.841
80	L	0.989	9.765	1.311
130	A	0.835	8.324	3.532
139	M	0.740	7.435	4.128
143	R	0.909	9.020	2.795
177	F	0.971	9.592	1.798
205	G	0.838	8.356	3.504
206	E	1.000**	9.861	0.916
212	D	0.806	8.059	3.742

Supplementary Table 2. Naïve Empirical Bayes results for positively selected sites in YopJ (*: P>95%; **: P>99%)

position	Residue	Prob (ω > 1)	Mean ω
**10**	I	0.852	3.09
**11**	S	0.998**	3.604
**20**	S	0.727	2.649

**33**	T	0.749	2.727
**40**	S	0.894	3.238
**52**	M	0.999**	3.611
**54**	V	0.759	2.76
**55**	E	0.742	2.701
**62**	I	0.833	3.022
**75**	L	0.791	2.874
**80**	L	0.888	3.216
**95**	R	0.765	2.783
**106**	G	0.802	2.913
**130**	A	0.79	2.869
**139**	M	0.686	2.503
**143**	R	0.83	3.011
**144**	T	0.776	2.822
**177**	F	0.995**	3.596
**185**	I	0.859	3.116
**189**	S	0.846	3.067
**205**	G	0.79	2.871
**212**	D	0.674	2.459
**242**	G	0.825	2.994
**243**	V	0.962*	3.479
**244**	G	0.839	3.043
**245**	T	0.998**	3.607

**246**	V	0.962*	3.479
**247**	V	0.931	3.368
**248**	N	0.979*	3.538
**251**	N	0.990**	3.58
**252**	E	0.699	2.547
**253**	T	0.861	3.122
**254**	I	0.999**	3.611
**255**	V	0.999**	3.608
**256**	N	0.912	3.302
**257**	R	1.000**	3.612
**258**	F	0.793	2.88
**259**	D	0.998**	3.607
**260**	N	0.979*	3.538
**261**	N	0.912	3.302
**262**	K	0.66	2.409
**263**	S	0.885	3.206
**264**	I	0.992**	3.584
**265**	V	1.000**	3.613

Supplementary Table S3. Primers used in this study

Primer	Sequence	Purpose
F yopJ	5'CAACAAGTTTCTCTACCGGAGAAT3’	screen for Δ*yopJ* allele
R yopJ	5'CTCATACCACCCGTACTCTAGCA3’	
yopJ F3	5’GATCGATATCCAAGTGCCCCCTAAGCCTTGAGTT3’	PCR clone *yopJ* orf into pACYC184, screen for *yopJ* point-mutant alleles
yopJ R2	5’GATCGTCGACCCCATACTGGAGCAAGATTTCC3’	
yopJ* F (sense)	5’GAAATGGATATTCAGCGAAGCTCATCTGAAGCTGGTATTTTTAGTTTTGCAC3’	mutate *yopJ*^{WT} to *yopJ*^{C172A}
yopJ* R (antisense)	5’GTGCAAAACTTAAAATACCAGCTTCAGATGAGCTTAGCTGAATATCCATTTC3’	
yopJ F4	5’GATCGCATGCCAAGTCCCCCCTAAGCCTTGAGTT3’	PCR clone *yopJ*^{C172A} into pCVD442
yopJ R4	5’GATCGCATGCCCCATACTGGAGCAAGATTTCC3’	
F177A F (sense)	5’GAAGCTCATCTGAATGTGGTATTTTTAGTTTGGCACTGGCAAAAAAAC3’	mutate *yopJ*^{C172A} to *yopJ*^{F177L}
F177A R (antisense)	5’GTTTTTTTGCCAGTGCCAAACTAAAAATACCACATTCAGATGAGCTTC3’	

[1]underlined sequence indicates restriction enzyme site used for cloning

Supplementary Figure 1

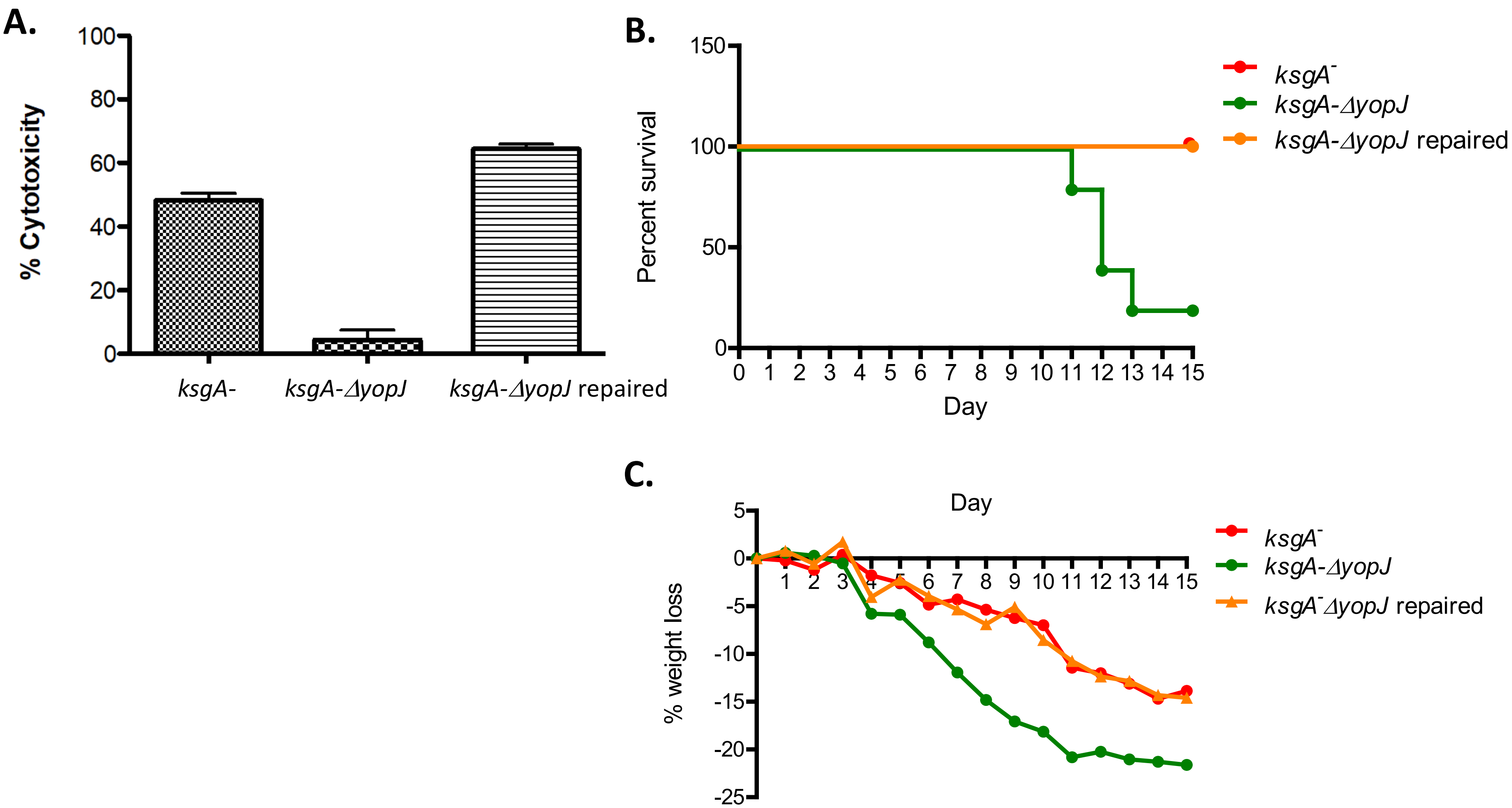

Supplementary Figure 2

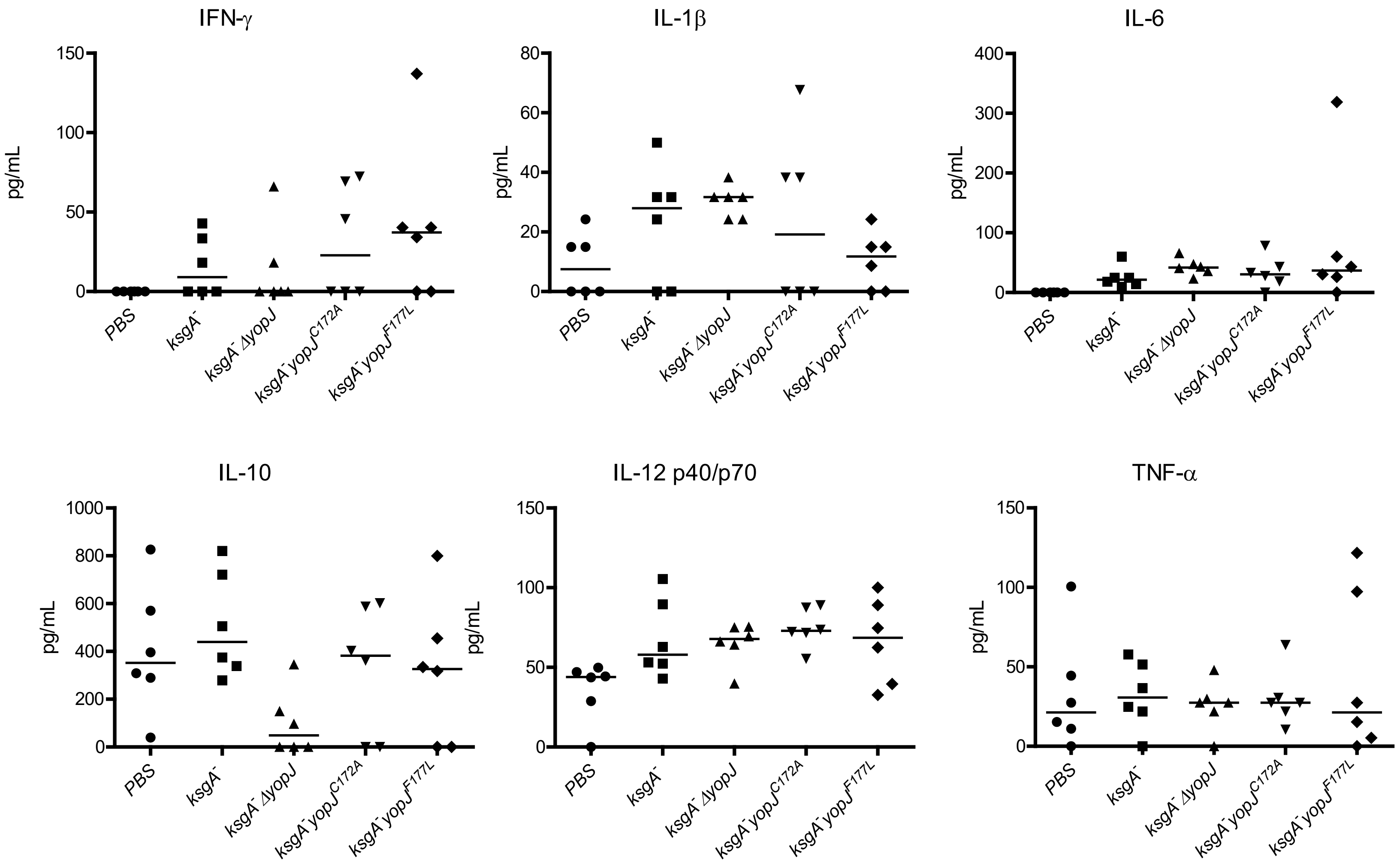

Supplementary Figure 3

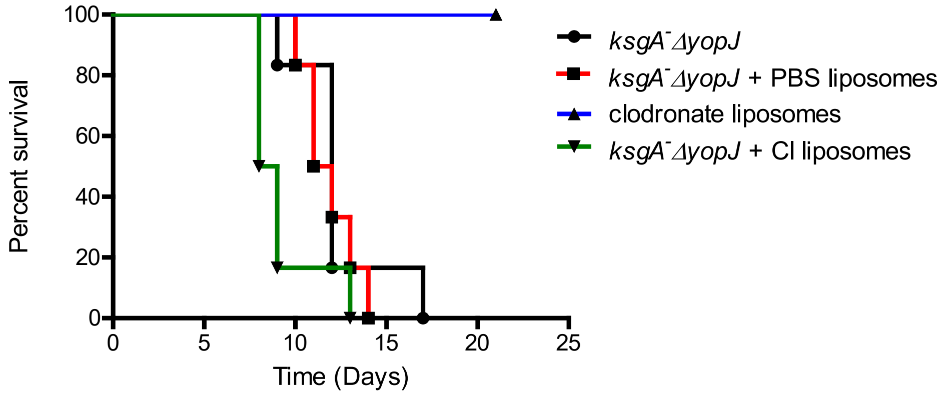

Supplement: Supplemental file 1 — Supplemental material. Download iai.00430-21-s0001.pdf, PDF file, 0.3 MB [file iai.00430-21-s0001.pdf]
